# Clinical interplay between autism spectrum disorder and bipolar disorder: a narrative review

**DOI:** 10.47626/2237-6089-2024-0939

**Published:** 2025-12-05

**Authors:** Mirela Paiva Vasconcelos-Moreno, Daniel Prates-Baldez, Júlio Santos-Terra, Iohanna Deckmann, Isabella Naomi Di Gesu, Renata de Sanson Lemann, Rudimar Riesgo, Carmem Gottfried, Flávio Kapczinski

**Affiliations:** 1 Laboratório de Psiquiatria Molecular Hospital de Clínicas de Porto Alegre Porto Alegre RS Brazil Laboratório de Psiquiatria Molecular, Hospital de Clínicas de Porto Alegre (HCPA), Porto Alegre, RS, Brazil.; 2 Universidade Federal do Rio Grande do Sul Programa de Pós-Graduação em Psiquiatria e Ciências do Comportamento Departamento de Psiquiatria e Medicina Legal Porto Alegre RS Brazil Programa de Pós-Graduação em Psiquiatria e Ciências do Comportamento, Departamento de Psiquiatria e Medicina Legal, Universidade Federal do Rio Grande do Sul (UFRGS), Porto Alegre, RS, Brazil.; 3 Instituto Nacional de Ciência e Tecnologia Translacional em Medicina Porto Alegre RS Brazil Instituto Nacional de Ciência e Tecnologia Translacional em Medicina (INCT-TM), Porto Alegre, RS, Brazil.; 4 Programa de Pós-Graduação em Ciências Biológicas: Bioquímica Porto Alegre RS Brazil Programa de Pós-Graduação em Ciências Biológicas: Bioquímica, UFRGS, Porto Alegre, RS, Brazil.; 5 Instituto Nacional de Ciência e Tecnologia em Neuroimunomodulação Instituto Oswaldo Cruz Fundação Oswaldo Cruz Rio de Janeiro RJ Brazil Instituto Nacional de Ciência e Tecnologia em Neuroimunomodulação (INCT-NIM), Instituto Oswaldo Cruz, Fundação Oswaldo Cruz, Rio de Janeiro, RJ, Brazil.; 6 Autism Wellbeing and Research Development Initiative BR-UK-CA Porto Alegre RS Brazil Autism Wellbeing and Research Development(AWARD), Initiative BR-UK-CA, Porto Alegre, RS, Brazil.; 7 Faculdade de Medicina UFRGS Porto Alegre RS Brazil Faculdade de Medicina, UFRGS, Porto Alegre, RS, Brazil.; 8 Programa de Pós-Graduação em Saúde da Criança e do Adolescente UFRGS Porto Alegre RS Brazil Programa de Pós-Graduação em Saúde da Criança e do Adolescente, UFRGS, Porto Alegre, RS, Brazil.; 9 Pró-Reitoria de Pesquisa UFRGS Porto Alegre RS Brazil Pró-Reitoria de Pesquisa, UFRGS, Porto Alegre, RS, Brazil.

**Keywords:** Autism spectrum disorder, bipolar disorder, comorbidity, overlapping symptoms

## Abstract

**Objective:**

Autism spectrum disorder (ASD) and bipolar disorder (BD) pose significant diagnostic challenges due to their clinical complexity. This review aims to examine the interface and overlapping features of these conditions, with a particular focus on the challenges associated with their comorbidity.

**Methods:**

We conducted a narrative review to examine clinical overlap, common psychiatric comorbidities, and a shared neurobiological basis between ASD and BD.

**Results:**

There is a notable convergence of symptoms in ASD and BD, including mood instability and emotional dysregulation; irritability, impulsivity, and aggressive behavior; deficits in social skills and social cognition; impairments in executive functions; sleep disturbances; problematic sexual behaviors; and sensory sensitivities. Common psychiatric comorbidities and shared neurobiological basis further underscore this potential interplay.

**Conclusion:**

Despite distinct clinical trajectories and diagnostic criteria, our findings indicate a significant overlap in symptoms and clinical presentations between ASD and BD. This complexity makes it challenging to identify the co-occurrence of ASD and BD, which can lead to difficulties in accurately diagnosing and managing both conditions simultaneously.

## Introduction

Autism spectrum disorder (ASD) is characterized by early-onset difficulties in social communication, along with markedly restricted, repetitive behaviors and interests. It affects individuals across the lifespan, with symptoms typically emerging in early childhood. Early diagnosis is often more readily made in individuals with severe symptoms and evident neurodevelopmental delays.^1^ However, in some cases, ASD may remain undiagnosed until adulthood, despite significant clinical impairment.^2^ The prevalence of ASD has been steadily increasing, with recent estimates indicating that approximately 1 in 36 children in the United States are affected,^3^ and around 1% of the global population.^4^ These prevalence rates underscore the importance of ASD as a major public health concern and highlight the need for accurate and timely diagnosis.

Bipolar disorder (BD), characterized by fluctuating mood episodes, is also a prevalent psychiatric condition affecting approximately 2% of the global population.^5^ Individuals typically experience their first manic episode in late adolescence or early adulthood. Diagnosis of BD involves a thorough clinical assessment, focusing on the presence, duration, and impact of mood episodes, and may also consider associated functional, social, and occupational impairments.^6^

The coexistence of undiagnosed ASD in adults and BD represents a complex clinical scenario that poses significant challenges for both diagnosis and treatment. Diagnostic overshadowing in ASD occurs when co-existing conditions or preconceived notions about ASD stereotypes obscure the recognition of ASD symptoms, leading to delayed or inaccurate diagnoses. Compounding this issue, both disorders share overlapping symptoms, such as emotional dysregulation, impulsivity, and social skills deficits. This symptomatic overlap leads to misdiagnosis, particularly in individuals presenting both ASD and BD.^7^ The challenge is to differentiate, on a case-by-case basis, whether these overlapping symptoms represent a single disorder with a broad presentation, the co-occurrence of both ASD and BD, or another condition altogether.

The primary aim of this narrative review is to explore the clinical interplay between BD and ASD, with a particular focus on level 1 ASD. This review will seek to elucidate the symptom overlap, highlight the unique diagnostic complexities encountered by clinicians, and underscore the need for improved strategies to enhance diagnostic accuracy. Additionally, it examines common comorbidities and explores shared neurobiological underpinnings between ASD and BD.

## Methods

We conducted a narrative literature review using MEDLINE (PubMed) with the search terms "Autism," "Autism Spectrum Disorder," "ASD," and "Bipolar Disorder." This review aimed to examine relevant clinical and preclinical studies, as well as observational and interventional trials, published up to April 2024. Articles were selected based on their relevance to the topic, without predefined inclusion or exclusion criteria, as this was not a systematic review.

## Results and discussion

### Clinical overlap between ASD and BD

**Figure 1:**
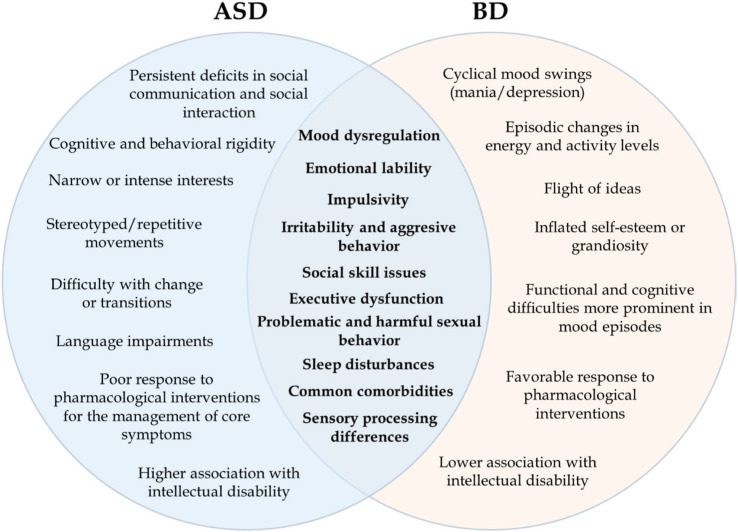
Symptom overlap and differentiation of symptomatology in autism spectrum disorder (ASD) and bipolar disorder (BD). The diagram highlights shared symptoms in the overlapping section, while unique characteristics of each condition are presented in the outer sections, aiding in distinguishing between the two disorders.

Below, we examine the overlapping symptoms of ASD and BD. Figure 1 provides an overview of their key similarities and differences.

Persistent deficits in social communication and social interaction are core features of ASD, while impairments in social cognition may also be present in BD. Cognitive and behavioral rigidity, narrow or intense interests, stereotyped or repetitive movements, difficulty with change or transitions, and language impairments are more specific symptoms of ASD, considering the DSM-5-TR diagnostic criteria.^6^ A poor response to pharmacological interventions for managing core symptoms, along with a higher association with intellectual disability (ID), are additional factors that help distinguish ASD from BD.

Mood instability, emotional dysregulation, impulsivity, irritability, and aggressive behavior are commonly observed in both ASD and BD. Impairments in executive functions are also observed in both ASD and BD, manifesting as common difficulties in planning and decision-making. Problematic sexual behaviors, sleep disturbances, and sensory processing alterations add an additional layer of complexity to the overlapping symptoms of these conditions. Cyclical mood swings, episodic changes in energy and activity levels, flight of ideas, inflated self-esteem or grandiosity, and functional and cognitive difficulties that are more prominent in mood episodes are specific symptoms of BD, according to DSM-5-TR.

#### Mood instability and emotional dysregulation

BD is primarily characterized by distinct periods of abnormal mood, alternating between depressive and manic episodes. Although ASD is defined by lifelong persistent symptoms, individuals with ASD often exhibit heightened sensitivity to environmental stimuli and changes in routine, as well as disruptive behavior when frustrated.^8,9^ In all these situations, individuals with ASD may frequently experience mood swings, which can mimic or complicate the identification of a comorbid affective disorder.^10,11^ On the other hand, mood instability in BD can also be influenced by environmental factors, including stress, life events, frustrations and disruptions in sleep patterns, further complicating the differentiation or identification of the simultaneous presence of these two conditions.^12^

Emotional dysregulation, characterized by difficulties in managing and responding to emotional experiences, is commonly observed in both ASD and BD, although the underlying mechanisms and triggers may differ. Individuals with ASD may exhibit emotional lability due to difficulties in recognizing, labeling, and expressing emotions, which can result in sudden and intense emotional responses.^10^ When individuals with ASD experience disruptions in their routines, they may exhibit symptoms that resemble manic or hypomanic episodes, such as psychomotor agitation, irritability, and distractibility. Some individuals with ASD may also experience abrupt mood fluctuations without an apparent cause.^10,11^

Similarly, individuals with BD may exhibit inadequate emotional reactivity or intense emotional expression, with emotional dysregulation occurring more frequently, but not exclusively, during mood episodes.^12^ Additionally, there is evidence suggesting that emotional instability may be a predictor of a future BD diagnosis.^13^ The presence of emotional dysregulation in both ASD and BD further complicates the differential diagnosis process and underscores the importance of assessing the context and temporal patterns of mood disturbances. Individuals with comorbid ASD and BD may face additional challenges due to the interaction between mood dysregulation and social communication difficulties inherent in ASD.

#### Irritability, impulsivity and aggressive behavior

Irritability, impulsivity, and aggressive behaviors are common manifestations in both ASD and BD. In individuals with ASD, increased irritability may be triggered by sensory overload, changes in routine, or difficulties with communication and social interactions.^6,14^ Adults with ASD might also exhibit periods of increased social intrusiveness, restlessness, and verbal aggression,^7^ traits that overlap with those observed in manic episodes.^8^ Likewise, individuals with BD frequently experience irritability, particularly during manic, depressive, or mixed episodes, often accompanied by impulsive and aggressive behaviors.^6^ Moreover, a recent systematic review indicated that impulsive behavior in BD can persist even during periods of euthymia, suggesting that impulsivity may be a trait feature of the disorder rather than solely a mood state.^15^

#### Deficits in social cognition and social skills

Social cognition is a complex psychological domain crucial for successful social interactions, as it supports adaptive social behaviors. Impairments in social communication and interaction are well-established core features of ASD.^6^ Individuals with ASD often struggle with nonverbal communication, social reciprocity, and maintaining peer relationships, and frequently exhibit problematic behaviors such as irritability and heightened emotional reactivity.^16^ They commonly face difficulties in understanding social cues and engaging at a level of social maturity comparable to their peers, which significantly impacts their ability to navigate social interactions and form meaningful connections.^17^ Furthermore, deficits in the theory of mind domain consequently affect interpersonal effectiveness, which is the ability to convey emotions and needs, as well as interact with others and build relationships based on reciprocal empathy.^16^ Patients with ASD may also use camouflage – a strategy to appear non-impaired – which can lead to depression and further obscure underlying social cognition deficits.^18^

Deficits in social cognition are also evident in BD, affecting individuals not only during symptomatic episodes but also throughout periods of euthymia.^19^ Individuals with BD exhibit moderate impairments in theory of mind, including difficulties with social perception and deficits in facial emotion recognition.^20-22^ Although less evident in BD than in ASD, recognizing that social skill impairments may be present provides important information for clinicians in an assessment context.

#### Deficits in executive functions

Executive functioning refers to a set of cognitive processes crucial for goal-directed behavior, attentional control, and self-regulation.^23^ Both ASD and BD are associated with deficits in executive functioning, including impairments in planning, organizing, decision-making, inhibitory control, set-shifting, verbal fluency, working memory, and problem-solving.^24,25^ These shared cognitive impairments may contribute to difficulties in decision-making, impulse control, and adaptive functioning observed in both conditions.^24,25^

#### Problematic and harmful sexual behavior

Individuals with ASD often encounter challenges related to sexuality due to impairments in social communication, sensory processing, and understanding of social norms.^26^ These challenges can limit sexual knowledge, reduce opportunities for appropriate experiences, and increase the risk of inappropriate behaviors. Given their social communication difficulties and behavioral rigidity, individuals with ASD may struggle to interpret complex social cues in intimate or sexual contexts. Some individuals with ASD adopt strategies to gain acceptance or inclusion within their social groups by engaging in sexual behaviors, which may also lead to periods of intensified sexual activity.^27^ Consequently, this group may experience feelings of exploitation or abuse in their sexual encounters, underscoring the distinct challenges each group faces in navigating aspects of sexual functionality.

In BD, mood swings significantly impact sexual functioning. Depressive episodes often lead to a decreased interest in sexual activity, while manic states can result in heightened sexual arousal.^28^ Manic or hypomanic episodes may lead to hypersexuality and risky sexual behaviors, such as unprotected sex, increased number of sexual partners, and disruption of daily routines.^29^ As individuals transition back to euthymia, they may experience emotions like feelings of exposure, exploitation or abuse, upon recognizing the risks associated with their behaviors during manic or hypomanic states. Overall, sexual behavior should be considered a relevant aspect of clinical care in both ASD and BD.

#### Sleep disturbances

Sleep disturbances are frequently reported in both ASD and BD and can significantly impact mood regulation. Individuals with ASD often experience difficulties initiating and maintaining sleep, which can exacerbate emotional dysregulation.^30^ Similarly, insomnia or hypersomnia are common in BD, occurring not only during mood episodes, but also during periods of euthymia. A multicenter longitudinal study involving 556 euthymic BD patients found that disrupted sleep disturbances may occur and could signal a more severe course of illness.^31^ Addressing sleep disturbances is crucial in managing mood dysregulation in both conditions.

#### Sensory processing differences

Sensory processing differences refer to variations in how the brain interprets and responds to sensory input, resulting in hyperreactivity or hyporeactivity. For example, individuals with ASD may exhibit extreme responses to specific auditory or tactile stimuli, engage in excessive smelling or touching objects, or display diminished sensitivity to pain, temperature, or other sensory experiences.^6,32^ Like individuals with ASD, those with BD may experience hyperreactivity to sensory input, often during manic or depressive episodes, typically in a less intense form.^33^

### Common psychiatric comorbidities in ASD and BD

When discussing the common interface between ASD and BD, it is essential to consider the high comorbidity rates between the two, as well as the presence of distinct comorbid conditions within each disorder. The association between these conditions was first noted in family-based studies, which provided clinical evidence supporting a potential link. Relatives of individuals diagnosed with ASD have been found to show a higher prevalence of affective disorders.^34^ Additionally, previous cohort studies have consistently identified the presence of BD in first-degree relatives as a significant risk factor for ASD.^35^

Some studies suggest that the prevalence of BD in individuals with ASD ranges from 6 to 21.4%.^35^ Although these data vary widely, they remain significantly higher than the prevalence of BD in the general population (2%).^36^ In a cohort study from Minnesota, for instance, individuals with ASD showed a cumulative incidence of BD of 7.3% by age 30, compared to 0.9% in the control group.^37^ Selten et al. conducted a population-based study demonstrating that individuals with ASD have an increased risk of developing non-affective psychotic disorders, schizophrenia, and BD compared to age- and sex-matched controls from the general population (OR 8.5 for BD before age 28 years).^38^ Importantly, individuals with ASD exhibited a significantly higher risk for these disorders compared to their non-ASD siblings.^38^ Sibling analyses in the study suggest that shared familial factors do not fully explain this association, indicating that ASD may independently play an important role in the development of BD.^38^

Both ASD and BD are linked to an increased risk of developing other psychiatric disorders, including greater vulnerability to suicidal ideation and behaviors.^39,40^ Individuals with ASD frequently experience elevated levels of symptoms and psychological distress throughout adulthood; in one sample, up to 79% met criteria for at least one psychiatric disorder during their lifetime.^41^

Meng-Chuan Lai et al. conducted a comprehensive meta-analysis examining the prevalence of co-occurring mental health diagnoses among individuals with ASD.^30^ The study reported prevalence rates of 28% for attention-deficit/hyperactivity disorder (ADHD), 20% for anxiety disorders, 13% for sleep-wake disorders, 11% for depressive disorders, 9% for obsessive-compulsive disorder (OCD), and 5% for BD.^30^ Conversely, individuals diagnosed with BD often present with one or more comorbid psychiatric conditions, such as ADHD, anxiety disorders, OCD, substance use disorder (SUD).^42,43^

Regarding its cognitive profile, ASD is characterized by high heterogeneity, ranging from intellectual disability (ID) to high intelligence.^44,45^ Individuals with higher cognitive abilities may display heightened sensitivities and intensities, often linked to perfectionism, rumination, and increased anxiety.^46^ On the other hand, ASD is associated with a higher prevalence of ID compared to the general population. Together, these cognitive extremes may influence symptom manifestation, diagnosis, and the management of comorbid BD. Population-based studies have found that BD is diagnosed four times more frequently in autistic individuals without ID than in those with ID.^38^ The reason for this difference remains unclear, but it may stem from the underdiagnosis of psychiatric disorders, as comorbid ID further impairs emotional expression and symptom reporting.^38^ Additionally, the increased risk of BD observed in individuals with ASD and higher cognitive abilities may be linked to greater awareness of their impacts.^38^

SUD can also co-occur in ASD and BD, potentially worsening mood dysregulation, impulsive behaviors, and social functioning difficulties.^47,48^ Although ASD was once thought to be protective against SUD, recent evidence indicates a higher prevalence among individuals with ASD – particularly those with psychiatric comorbidities and those not receiving psychotropic medication.^49^ SUD can likewise mask core symptoms, complicating accurate diagnosis in individuals with comorbid ASD and BD. Furthermore, certain behaviors associated with ASD and BD can be misinterpreted as personality disorders or may coexist with them.^6^ The reviewed literature highlighted that individuals with borderline personality disorder typically score higher on assessments measuring autistic traits compared to non-clinical populations.^50^ Additionally, adults diagnosed with level 1 ASD may exhibit comorbidities or behaviors resembling schizotypal, schizoid, or narcissistic traits. Similarly, individuals with type I BD frequently present with comorbid personality disorders, particularly schizotypal and borderline personality disorder.^6^

In sum, comorbidities between ASD and BD can contribute to a range of psychosocial challenges and symptom complexities, and may result in additional difficulties in educational settings, occupational functioning, and social relationships.^48,51^ These findings underscore the need for tailored mental health care and emphasize the importance of adequate treatment for individuals with ASD and BD.

### Shared neurobiological basis between ASD and BD

Understanding the biological origins of ASD and BD may help clarify the commonalities and complexities observed in these conditions. The connection between ASD and BD is likely influenced by shared genetic factors, as demonstrated in previous whole-genome studies.^52^ A growing body of research supports this, identifying genetic variants and chromosomal regions associated with both conditions, suggesting overlapping genetic mechanisms.^53,54^

The etiologies of ASD and BD remain incompletely understood; however, the role of genetics in these conditions has become increasingly established. Recent meta-analyses of twin studies on ASD have estimated heritability to be between 64 and 91%, with little to no significant contribution from shared environmental factors.^55^ BD, on the other hand, is also highly heritable and shows genetic overlap with other psychiatric disorders.^53,56,57^

Investigating the genetic associations between psychiatric disorders, a study using the GeneAnalytics program identified 23 genes shared by ASD, BD, and schizophrenia.^56^ These genes are involved in nine major biological pathways, including circadian entrainment, and affect dopamine and serotonin regulation, as well as signal transduction pathways, influencing mood, behavior, and physical activity levels.^56^ Recent evidence points to common expression patterns of risk genes associated with these mental illnesses, converging in specific cortical regions and suggesting a shared vulnerability of the cortex to transcriptional dysregulation across these disorders.^57^ Additionally, epigenetic mechanisms, which regulate gene expression without altering DNA sequence, may also contribute to the overlap between ASD and BD.^58-60^

### Diagnostic challenges in ASD and BD

As discussed in this article, there is a significant convergence of features between ASD and BD, which can contribute to diagnostic overshadowing. Recognizing this intersection is crucial, not only to distinguish between the two conditions, but also to acknowledge their potential coexistence. Individuals presenting with overlapping features may face challenges in the accurate identification and effective management of both disorders. Therefore, it is essential to highlight the specific diagnostic challenges that hinder recognition of their comorbidity.

An initial consideration is the age of onset and developmental stage, which can add complexity to the diagnostic process. While ASD is typically identified in early childhood, the onset of BD often occurs later and may be delayed or obscured in individuals with ASD. This can result in misdiagnosis or a failure to recognize emerging mood symptoms, further complicating timely and accurate diagnosis.^61^ In contrast, longitudinal studies have shown that individuals with comorbid ASD and BD often exhibit an earlier onset of mood symptoms compared to those with BD alone.^62^

Moreover, the clinical presentations of ASD and BD can evolve over time, adding further complication to the diagnostic process. Symptom patterns may shift or intensify across different developmental stages or in response to environmental triggers, such as stressful life events or challenging transitions to adulthood.^41^ In some cases, level 1 may go undetected during childhood and may only be recognized when adolescents seek treatment for suspected depressive symptoms. Under these circumstances, mood symptoms often serve as the primary reason for medical evaluations, leading to the subsequent identification of a comorbid ASD diagnosis. Youth with comorbid BD and ASD often show mixed or atypical mood symptoms such as distractibility, racing thoughts, and blunted emotional reactivity, along with greater functional impairment and social withdrawal.^62^ These presentations may be less readily recognized as classic BD, particularly early in the illness, and can obscure the onset of BD.

Despite the distinct diagnostic criteria for ASD and BD outlined in the DSM-5-TR and other classification systems, symptoms such as social withdrawal, irritability, and mood lability can blur distinctions and contribute to diagnostic overshadowing.^6^ Diagnostic confusion can occur in both directions: unrecognized BD may be masked by ASD, while undiagnosed ASD may be misinterpreted as a mood disorder. Social communication difficulties in ASD can hinder the recognition of BD symptoms, as individuals with ASD often struggle to articulate or express their mood states and internal experiences. These challenges may obscure mood symptoms, complicating diagnosis and increasing the risk of underdiagnosing BD. Conversely, core features of ASD – such as social withdrawal and difficulty expressing emotions – may be mistaken for depressive symptoms.^61^ The UK National Institute for Health and Care Excellence (NICE) guidelines recommend considering an autism assessment in adults who exhibit possible autistic traits according to psychiatric diagnostic criteria, along with at least one of the following: (1) difficulty securing or maintaining employment or education; (2) challenges in forming or sustaining social relationships; (3) current or past involvement with mental health or learning disability services; or (4) a history of neurodevelopmental (such as ADHD) or psychiatric disorders.^63^ These guideline items serve as a practical tool to identify adults who may benefit from further autism assessment. ASD diagnosis is primarily clinical, requiring multiple information sources, though standardized tools like the Autism Diagnostic Observation Schedule (ADOS) and the Autism Diagnostic Interview-Revised (ADI-R) are considered gold standards and reliably support adult diagnosis.^6,64^

Identifying comorbidity between ASD and BD is crucial, as recent research shows that clinicians often focus on symptoms of other mental health or neurodevelopmental disorders, like ADHD, which can lead to overlooking or underestimating ASD symptoms – especially in individuals with mild or atypical presentations.^65^ Stereotypes that associate ASD primarily with males and specific behaviors may further contribute to missed diagnoses in females or individuals from diverse backgrounds who exhibit different symptom profiles.

## Conclusion

Despite distinct clinical trajectories and diagnostic criteria, this narrative review highlights the overlap of symptoms and clinical aspects, common comorbidities, and shared neurobiological basis between ASD and BD. Identifying the comorbidity between ASD and BD is essential, as it can alter the clinical presentation of both disorders and, if unrecognized, may lead to misdiagnosis, delayed intervention, and poorer outcomes. Understanding these overlapping symptoms is crucial for clinicians to improve their knowledge and approach to managing these complex comorbid conditions.

## Data Availability

Not applicable.
